# Oligomeric amyloid *β* induces IL-1*β* processing via production of ROS: implication in Alzheimer's disease

**DOI:** 10.1038/cddis.2013.503

**Published:** 2013-12-19

**Authors:** B Parajuli, Y Sonobe, H Horiuchi, H Takeuchi, T Mizuno, A Suzumura

**Affiliations:** 1 Department of Neuroimmunology, Research Institute of Environmental Medicine, Nagoya University, Furo-cho, Chikusa-ku, Nagoya 464-8601, Japan

**Keywords:** microglia, oligomer A*β*, NLRP3, IL-1*β*

## Abstract

Alzheimer's disease (AD) is a chronic neurodegenerative disease characterized by progressive neuronal loss and cognitive decline. Oligomeric amyloid *β* (oA*β*) is involved in the pathogenesis of AD by affecting synaptic plasticity and inhibiting long-term potentiation. Although several lines of evidence suggests that microglia, the resident immune cells in the central nervous system (CNS), are neurotoxic in the development of AD, the mechanism whether or how oA*β* induces microglial neurotoxicity remains unknown. Here, we show that oA*β* promotes the processing of pro-interleukin (IL)-1*β* into mature IL-1*β* in microglia, which then enhances microglial neurotoxicity. The processing is induced by an increase in activity of caspase-1 and NOD-like receptor family, pyrin domain containing 3 (NLRP3) via mitochondrial reactive oxygen species (ROS) and partially via NADPH oxidase-induced ROS. The caspase-1 inhibitor Z-YVAD-FMK inhibits the processing of IL-1*β*, and attenuates microglial neurotoxicity. Our results indicate that microglia can be activated by oA*β* to induce neuroinflammation through processing of IL-1*β*, a pro-inflammatory cytokine, in AD.

Alzheimer's disease (AD) is a chronic neurodegenerative disease characterized by progressive cognitive decline.^1, 2, 3^ The pathological hallmarks of AD are neuronal loss, neurofibrillary tangles, accumulation of activated glial cells, gliosis, and the deposition of amyloid that forms senile plaques. The imbalance between the production of amyloid *β* (A*β*), the proteolytic fragment of amyloid precursor protein (APP), and its clearance is considered to be central event in the pathogenesis of AD.^4, 5^ The A*β* peptide undergoes transition from monomer to oligomer and then forms insoluble fibrillar A*β* (fA*β*), which is the major component of senile plaque.^6, 7^ Although fA*β* was thought to be the primary entity responsible for AD, recent evidence suggests that soluble oligomeric amyloid *β* (oA*β*) found in the cortex of AD patients contributes to the pathogenesis of AD.^8^ Indeed, the level of oA*β* in the AD brain or cerebrospinal fluid is directly correlated with the degree of synaptic loss and severity of cognitive decline.^9, 10^

Inflammatory process initiated by activated microglia is another essential component of AD.^11^ Accumulation of activated microglia is observed around degenerating neurons. It has been shown that microglial activation precedes cognitive decline and the formation of senile plaque in different APP transgenic mice, animal models of AD.^12, 13, 14^

Interleukin (IL)-1*β*, a member of the IL-1 cytokine family, is produced as the inactive precursor pro-IL-1*β* in the cytoplasm in response to a wide variety of stimuli.^15, 16^ In order to exert its functions, pro-IL-1*β* must be processed into its mature active form by the protease caspase-1, which itself is activated by cytosolic multiprotein complexes called inflammasomes.^17, 18^ NOD-like receptor family, pyrin domain containing 3 (NLRP3) is the most intensively studied inflammasome complex protein and undergoes bipartite activation in macrophage and microglia.^17^ The first signal, usually microbial toxins-like lipopolysaccharides (LPS), induces NLRP3 and pro-IL-1*β* expression. The second signal, usually many unrelated entities like urate, extracellular ATP, and fA*β*, induces NLRP3 oligomerization with the adapter protein apoptosis-associated-speck-like protein (ASC), which leads to autocatalytic activation of caspase-1. This activation of caspase-1 requires an efflux of potassium (K^+^).^19^ Activated caspase-1 then processes pro-IL-1*β* to mature IL-1*β*.^20, 21, 22, 23^ In addition, NLRP3 activation in microglia is reported to contribute to the progression of AD-like pathology in APP/PS1 transgenic mice, and NLRP3 knock out (KO) mice are reported to have decreased disease burden.^24^ Although oA*β* is postulated to activate inflammasomes,^25^ how oA*β* induces NLRP3 activation to process pro-IL-1*β* to the mature form remains unknown. Here we show that oA*β* increases the processing of pro-IL-1*β* into mature IL-1*β* in microglia via reactive oxygen species (ROS)-dependent activation of NLRP3.

## Results

**Figure 1:**
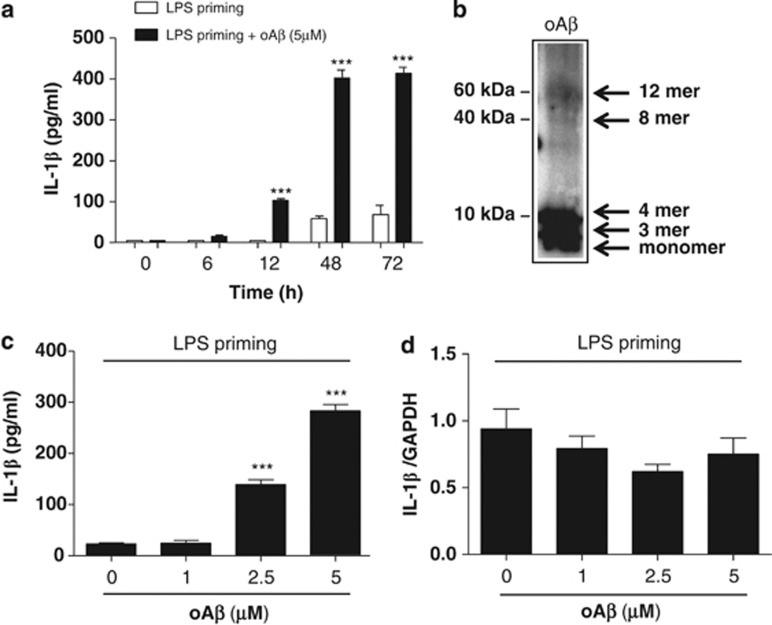
oA*β* induces IL-1*β* release in LPS-primed microglia. Microglia primed with LPS for 3 h were washed with ice-cold PBS and treated with oA*β* for varying times; the concentration of IL-1*β* in the culture supernatant was then measured (**a**). ***P<0.001, *versus* 0 h. Western blot analysis of oA*β* used in the present study (**b**). The blot was incubated with mouse anti oA*β* monoclonal antibodies (6E10) (1 : 1000, Chemicon). LPS-primed microglia were treated with oA*β* for 48 h, and the concentration of IL-1*β* in the culture supernatant as well as mRNA expression were measured by ELISA (**c**) and qPCR (**d**). Data indicate means±S.D. for five independent experiments. ***P<0.001, *versus* LPS-primed microglia

**Figure 2:**
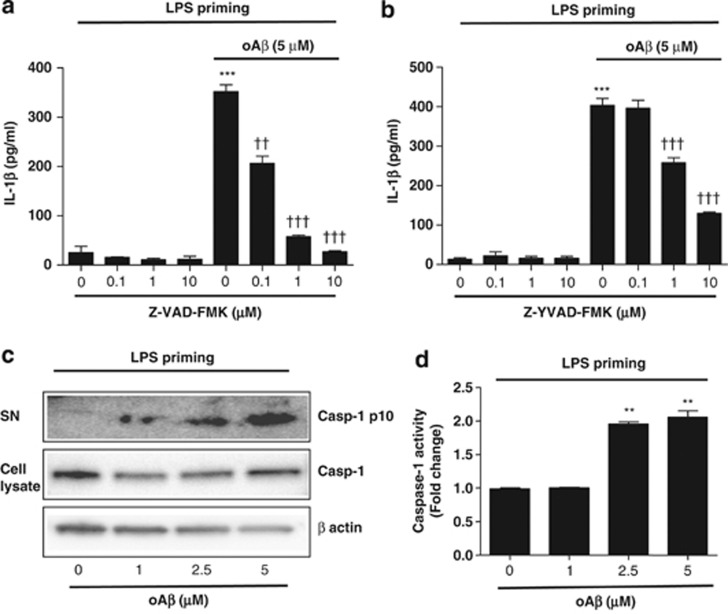
oA*β* induces IL-1*β* secretion/release via caspase-1 activation. LPS-primed microglia were treated with Z-VAD-FMK (**a**) or Z-YVAD-FMK (**b**) for 30 min before oA*β* stimulation, and IL-1*β* in the culture supernatant was measured at 48 h. Data indicate means±S.D. for four independent experiments. ***P<0.001, *versus* LPS-primed microglia as control (ctl). ††, or ††† denotes *P*<0.01, or 0.001, respectively, *versus* LPS-primed microglia+oA*β*. (**c**) After LPS-priming microglia were treated with oA*β* for 48 h, Casp-1 p10 in the culture supernatant as well as caspase-1 and *β*-actin in the cell lysates were assessed by western blotting. Data are representative of two independent experiments. (**d**) LPS-primed microglia were treated with oA*β* for 48 h, and caspase-1 activity was measured. Data indicate means±S.D. for three independent experiments. **P<0.01, *versus* LPS-primed microglia without oA*β*

We first assessed whether oA*β* induces IL-1*β* mRNA or processes IL-1*β* protein in microglia. We found that oA*β* alone did not induce IL-1*β* mRNA and protein in microglia (Supplementary Figures 1a and b). To assess whether oA*β* affects the processing of IL-1*β*, we transiently activated microglia with LPS (1 *μ*g/ml) for 3 h (LPS priming). The cells were then washed twice with ice-cold PBS and further stimulated with oA*β* (5 *μ*M) for varying times (0–72 h), and IL-1*β* concentration in the culture supernatant was measured. We found that oA*β* time-dependently increased IL-1*β* concentration in the culture supernatant when compared with transiently activated microglia with LPS for 3 h, which served as control (Figure 1a). Western blot analysis of oA*β* used in the present study was shown in Figure 1b. In addition, oA*β* dose-dependently increased IL-1*β* secretion (Figure 1c). As oA*β* alone did not upregulate mRNA levels of IL-1*β* (Figure 1d), these results indicate that oA*β* upregulates processing of IL-1*β* in LPS-primed microglia. As pro-IL-1*β* is reported to be processed by a caspase-dependent pathway.^15^ To determine whether oA*β*-induced IL-1*β* secretion is dependent on caspase, microglia primed with LPS for 3 h were treated with the pan-caspase inhibitor Z-VAD-FMK or caspase-1 inhibitor Z-YVAD-FMK for 30 min before oA*β* stimulation. We then measured IL-1*β* in culture supernatant at 48 h. Both Z-VAD-FMK and Z-YVAD-FMK dose-dependently decreased IL-1*β* secretion in the culture supernatant (Figures 2a and b). We next assessed the cleaved fraction of caspase-1 (Casp-1 p10) by western blotting and found that oA*β* dose-dependently increased the secretion of Casp-1 p10 in the culture supernatant (Figure 2c). Similarly, treatment of oA*β* after LPS priming dose-dependently increased caspase-1 activity in microglia (Figure 2d).

**Figure 3:**
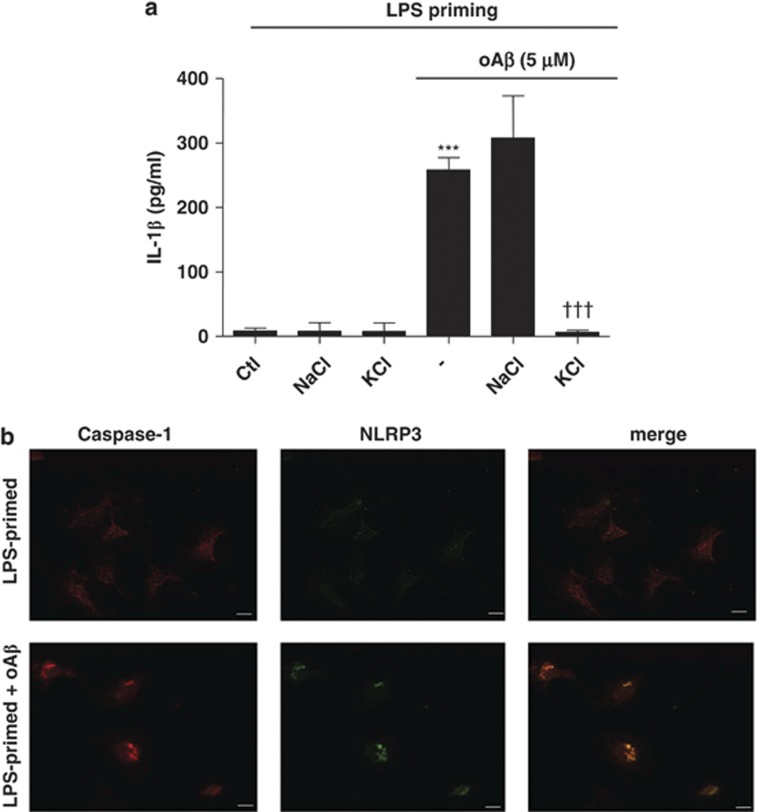
oA*β*-induced caspase-1 activation is dependent on NLRP3. (**a**) LPS-primed microglia were treated with NaCl or KCl before oA*β* stimulation, and IL-1*β* in the culture supernatant was measured at 48 h. Data indicate means±S.D. for four independent experiments. ***P<0.001, *versus* LPS-primed microglia (ctl). ^†††^*P*<0.001, *versus* LPS priming+oA*β*+KCl. (**b**) LPS-primed microglia were treated with oA*β* for 48 h, and co-localization of NLRP3 and caspase-1 were assessed by immunocytochemistry. Data are representative of three independent experiments. Scale bar represents 10 *μ*m

**Figure 4:**
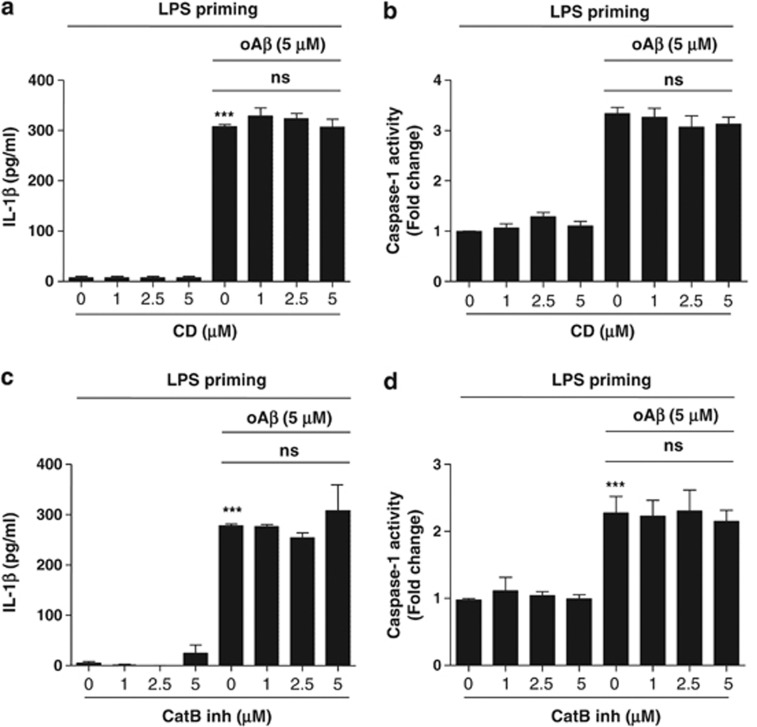
oA*β*-induced IL-1*β* release is independent on phagocytosis or cathepsin B. LPS-primed microglia were treated with cytochalasin D for 30 min before oA*β* stimulation, and IL-1*β* (**a**) in the culture supernatant as well as caspase-1 activity (**b**) were measured at 48 h. Data indicate means±S.D. for three independent experiments. ***P<0.001, *versus* LPS-primed microglia (ctl). ns indicates not significant. LPS-primed microglia were treated with cathepsin B inhibitor for 30 min before oA*β* stimulation, and IL-1*β* (**c**) in the culture supernatant as well as caspase-1 activity (**d**) were measured at 48 h. Data indicate means±S.D. for three independent experiments. ***P<0.001, *versus* LPS-primed microglia (ctl). ns indicates not significant

**Figure 5:**
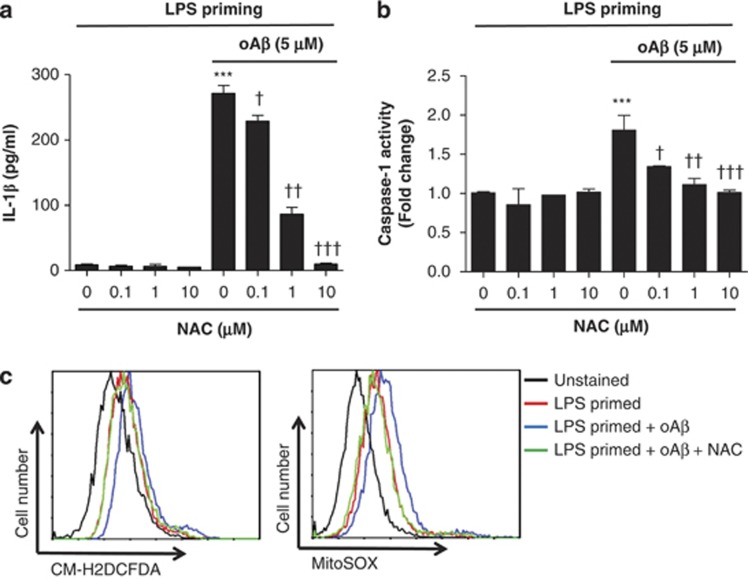
oA*β*-induced IL-1*β* release is dependent on ROS. LPS-primed microglia were treated with varying doses of NAC (0.1–10 *μ*M) for 30 min before oA*β* stimulation, and IL-1*β* (**a**) in the culture supernatant as well as caspase-1 activity (**b**) were measured at 48 h. Data indicate means±S.D. for three independent experiments. ***P<0.001, *versus* LPS-primed microglia (ctl). †, ††, or ††† denotes *P*<0.05, 0.01, or 0.001, respectively, *versus* LPS priming+oA*β*+NAC (**c**). LPS-primed microglia were treated with the ROS scavenger NAC (10 *μ*M) for 30 min before oA*β* stimulation and cellular (CM-H_2_DCFDA) and mitochondrial (MitoSOX) ROS were assessed by flow cytometry. Data are one representative of three independent experiments

**Figure 6:**
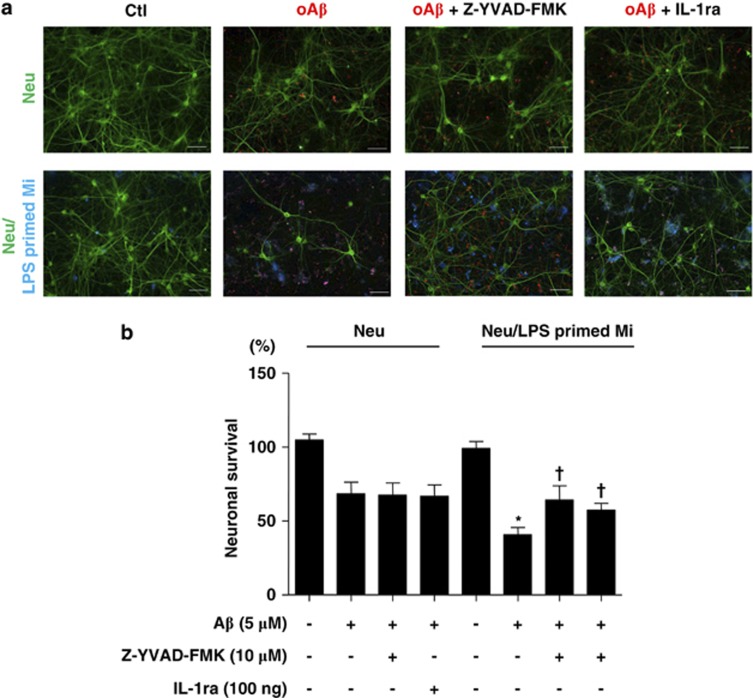
LPS-primed microglia treated with oA*β* induce neuronal cell death via caspase-1, and IL-1*β*. LPS-primed microglia were cocultured with primary cortical neurons. The cells were then treated with Z-YVAD-FMK or IL-1ra before oA*β* treatment. Neurons were stained with anti-MAP2 antibodies (green), microglia were stained with Cy-5-conjugated anti-CD11b antibodies (blue), and oA*β* was stained with anti-4G8 antibodies (red). Neuronal viability was assessed by MAP2 staining (**a** and **b**). Data indicate means±S.D. for three independent experiments. *P<0.05, *versus* neurons treated with oA*β*, ^†^*P*<0.05, *versus* neurons cocultured with LPS-primed microglia treated with oA*β*. Scale bar represents 50 *μ*m. Neu indicates neurons and Mi indicates microglia

To determine whether oA*β*-induced IL-1*β* processing is dependent on NLRP3, we increased the K^+^ concentration in the culture medium, which was previously described to inhibit NLRP3.^19^ We found that the increased K^+^ concentration, by the addition of KCl, significantly decreased IL-1*β* release from microglia (Figure 3a). The addition of NaCl did not affect IL-1*β* release. Furthermore, oA*β* stimulation induced the co-localization of caspase-1 with NLRP3 (Figure 3b). NLRP3 is reported to be activated by lysosomal destabilization and release of cathepsin B in response to phagocytosis.^22, 23, 26^ To evaluate the requirement of phagocytosis and cathepsin B release in oA*β*-induced IL-1*β* secretion, phagocytosis and cathepsin B were pharmacologically inhibited with cytochalasin D and cathepsin B inhibitor, respectively. We found that cytochalasin D inhibited fA*β*-induced IL-1*β* release and caspase-1 activity as previously described^22^ (Supplementary Figures 2a and b); however, it had no effect on oA*β*-induced IL-1*β* secretion and caspase-1 activity (Figures 4a and b). Similarly, cathepsin B inhibitor decreased fA*β*-induced IL-1*β* secretion and caspase-1 activity as previously described^22^ (Supplementary Figures 2c and d), it had no effect on oA*β*-induced IL-1*β* secretion and caspase-1 activity (Figures 4c and d). ROS are reported to act as danger signal for NLRP3 inflammasome activation.^21, 27, 28^ High concentrations of ROS inhibitors are reported to block NF-*κ*B-mediated by priming of NLRP3 inflammasome.^29^ We treated microglia with N-acetylcysteine (NAC), a potent ROS scavenger, for 30 min after LPS priming and before the addition of oA*β*. NAC dose-dependently decreased oA*β*-induced IL-1*β* secretion (Figure 5a). Similarly, NAC also inhibited oA*β*-induced caspase-1 activity (Figure 5b). Similarly, gp91ds-tat, an NADPH oxidase (NOX)-specific inhibitor, also dose-dependently decreased oA*β*-induced IL-1*β* secretion as well as caspase-1 activity, but not as potently as NAC (Supplementary Figures 3a and b). These results indicate that oA*β*-induced IL-1*β* secretion is partially dependent on NOX. Mitochondrial ROS are reported to activate NLRP3, so we next determined cellular and mitochondrial ROS production by flow cytometry. Microglia treated with oA*β* after LPS priming produced cellular and mitochondrial ROS, which were inhibited by NAC (Figure 5c). We also assessed whether LPS-primed microglia affect neuronal viability. LPS-primed microglia were cocultured with primary cortical neurons and treated with oA*β* (5 *μ*M) with or without Z-YVAD-FMK or IL-1ra. Neuronal cultures were also treated with oA*β* with or without Z-YVAD-FMK or IL-1ra. We found that treatment of neuronal cultures with oA*β* decreased the viability of neurons. The neuronal damage with oA*β* was further enhanced in the neurons/LPS-primed microglia cocultures. Although Z-YVAD-FMK or IL-1ra had no effect in the neuronal cultures, it attenuated microglia-induced neurotoxicity in the neuron/LPS-primed microglia cocultures (Figures 6a and b).

## Discussion

Microglial-mediated neuroinflammation contributes to the pathogenesis of AD. Indeed, microglial activation and subsequent production of neurotoxic pro-inflammatory molecules have a pivotal role in the progression of AD. However, whether A*β*, a main component of misfolded protein in the AD brain, could induce the production of pro-inflammatory cytokines is controversial. It has been reported that oA*β* does not induce IL-1*β* mRNA in microglia.^30^ However, other reports indicate that oA*β* induces various inflammatory mediators such as IL-1*β*, TNF-*α*, and NO.^31, 32, 33^ In this study, we have shown that oA*β* alone is not sufficient to induce IL-1*β* mRNA or increase IL-1*β* secretion in unstimulated microglia. oA*β* induces IL-1*β* secretion via activation of caspase-1 when microglia are primed with toll-like receptor (TLR) 4 ligand LPS. We also showed that both mitochondrial as well as NOX2-induced ROS contribute to oA*β*-induced caspase-1 activation. Furthermore, oA*β* has been shown to induce ROS in microglia by activation of NOX and mitochondria damage.^34, 35, 36, 37, 38^ ROS are reported to activate caspase-1 via NLRP3.^27, 28^ ROS induce oxidation of K^**+**^ channel.^39^ Similarly, oA*β* is reported to induce pore formation in the cell membrane^40^ and to alter K^**+**^ current in neurons.^41^ Thus, oA*β*-induced pore formation or oxidation of K^**+**^ channel might lead to K^**+**^ efflux activating NLRP3 inflammasome in microglia. We have also shown that inhibition of K^**+**^ efflux decreases oA*β*-induced IL-1*β* secretion. K^**+**^ efflux is required for NLRP3 activation.^19^

Our results indicate that the mechanism of oA*β*-induced IL-1*β* secretion is different from that induced by fA*β*. oA*β*-induced IL-1*β* secretion by microglia was not dependent on phagocytosis and lysosomal disruption with subsequent release of cathepsin B, because we found that the inhibition of phagocytosis by cytochalasin D and cathepsin B inhibitors had no effect on IL-1*β* secretion. However, fA*β*-induced IL-1*β* secretion is dependent on phagocytosis with subsequent lysosomal disruption. oA*β* and fA*β* differentially activate microglia and neurons.^30, 42^ For instance, oA*β* is reported to inhibit phagocytosis, whereas fA*β* is reported to stimulate phagocytosis.^42^ Moreover, oA*β* is reported to be more neurotoxic than fA*β*.^30^ We have also shown that oA*β* induces far greater secretion of IL-1*β* than fA*β* in LPS-primed microglia. We further showed inflammasome activation in microglia increases oA*β*-induced neuronal cell death, which is ameliorated by the inhibition of caspase-1 and IL-1*β.* Consistent with this observation, genetic deletion of NLRP3 in mice expressing mutant human APP/PS1, an animal model of AD, deceases their disease burden.^24^

The role of IL-1*β* in AD pathology is complex. IL-1*β* transgenic mice expressing mutant human APP/PS1 are reported to have decreased plaque formation, although the total amount of oA*β* is unaltered.^43^ However, IL-1*β* transgenic mice are reported to have learning and memory impairment.^44^ IL-1*β* can also affect synaptic plasticity and inhibit long-term potentiation.^45, 46^ It has been shown that secreted mature IL-1*β* induces the phosphorylation of tau protein and mediates the formation of neurofibrillary tangles.^47, 48^ IL-1*β* can be elevated before the formation of amyloid plaque in patients with Down syndrome, who invariably develop AD-like pathology.^49^ Thus, oA*β*-induced IL-1*β* secretion by microglia may augment neuroinflammation, increase neuronal cell death, and contribute to the pathogenesis of AD. Indeed, the infusion of oligomeric human amyloid *β* in mice lacking IL-1 receptor antagonist (IL-1ra) induces microglial activation and causes neuronal cell death.^50^

In conclusion, our results indicate that oA*β* induces the secretion of active IL-1*β* via increased activation of caspase-1 in LPS-primed microglia, which is dependent on mitochondrial and NOX2-induced ROS production. Secreted IL-1*β* is involved in neuronal cell death that is ameliorated by inhibiting caspase-1 activation or by neutralization of IL-1*β*. Thus, the cascade of oA*β*-induced IL-1*β* secretion in microglia may be a target for treating AD.

## Materials and Methods

### Reagents

LPS, N-acetyl-L-cysteine (NAC) and cytochalasin D were obtained from Sigma-Aldrich (St. Louis, MO, USA). Z-VAD-FMK (pan-caspase inhibitor), Z-YVAD-FMK (caspase-1 inhibitor), and Ac-Leu-Val-lysinal (cathepsin B inhibitor) were obtained from Calbiochem (Gibbstown, NJ, USA). Anti-cryopyrin (sc-34410) and anti-caspase-1 antibodies (sc-514) were from Santa Cruz Biotechnology (Santa Cruz, CA, USA). Gp-91 ds tat was from Anaspec (Fremont, CA, USA). IL-1ra was obtained from R&D (Minneapolis, MN, USA).

### Cell culture

All animal experiments were conducted under protocols that were approved by the Animal Experiment Committee of Nagoya University. All primary cultures were prepared from C57BL/6 mice (Japan SLC, Hamamatsu, Japan).

Microglia were isolated from primary mixed glial cell cultures prepared from newborn mice on day 14 using the ‘shaking off' method as previously described.^51^ The purity of the cultures (>99%) was determined by anti-CD11b immunostaining (BD Biosciences, Franklin Lakes, NJ, USA). The cultures were maintained in Dulbecco's modified Eagle's minimum essential medium (Sigma-Aldrich) supplemented with 10% fetal bovine serum (SAFC Biosciences, Lenexa, KS, USA), 5 *μ*g/ml bovine insulin (Sigma-Aldrich) and 0.2% glucose.

Primary neuronal cultures were prepared from the cortices of mouse embryos at embryonic day 17 (E17) as described previously.^52^ Briefly, cortical fragments were dissociated into single cells in dissociation solution (Sumitomo Bakelite, Akita, Japan) and resuspended in nerve culture medium (Sumitomo Bakelite). Neurons were seeded onto 12-mm polyethyleneimine (PEI)-coated glass cover slips (Asahi Techno Glass Corp, Chiba, Japan) at a density of 5 × 10^4^ cells/well in 24-well plates. The purity of the culture was more than 95% as determined by NeuN-specific immunostaining (Merck Millipore, Billerica, MA, USA).

Neuron-microglia cocultures were prepared as follows: 1 × 10^5^ microglia in 100 *μ*l of neuronal medium were added to neuronal cultures (5 × 10^4^ neuronal cells) in 24-well plates on day 14.

### Preparation of oA*β* and fA*β*

oA*β* and fA*β* were prepared as previously described.^30^ To form fA*β* synthetic human A*β*1-42 (Peptide Institute, Osaka, Japan) was dissolved in 0.02% ammonia solution at a concentration of 250 *μ*mol/l, diluted to 25 *μ*mol/l in PBS, and incubated at 37 °C for 72 h. Briefly, oA*β*1-42 was prepared by dissolving A*β*1-42 to 1 mmol/l in 100% 1,1,1,3,3,3-hexafluoro-2-propanol. 1,1,1,3,3,3-Hexafluoro-2-propanol was dried by a vacuum desiccator and resuspended to 5 mmol/l in DMSO. To form oligomers, amyloid peptide was diluted to a final concentration of 100 μmol/l with Ham's F-12, incubated at 4 °C for 24 h, and then immediately added to cultures at a final concentration 5 μmol/l. Formation of oA*β* was confirmed by western blotting as previously described.^30^

### Measurement of IL-1*β* and caspase-1 activity

Microglia, seeded at a density of 1 × 10^5^ cells/well in 24-well plates, were treated with LPS for 3 h. The cells were then washed twice and treated with oA*β*. Supernatants were collected and the levels of IL-1*β* in culture supernatant were determined by ELISA according to the manufacturer's instruction (BD Biosciences). Microglia, seeded at a density of 1 × 10^7^ cells and treated as described above, were measured for caspase-1 activity according to the manufacturer's instruction (Merck Millipore).

### RT-PCR

For quantitative PCR, the total cellular RNA was extracted using the RNeasy Mini Kit (Qiagen, Hilden, Germany). cDNA was synthesized from total cellular RNA that was denatured for 5 min at 65 °C, followed by a reverse transcription reaction using the SuperScript II (Life Technologies, Carlsbad, CA, USA). The cDNA served as a template to amplify genes in quantitative PCRs with TaqMan Gene Expression assays (Applied Biosystems, Foster City, CA, USA), Universal PCR Master Mix (Applied Biosystems), and Rotor-Gene Q (Qiagen). Expression levels of target genes were calculated using a comparative method and normalization to GAPDH expression levels as previously described.^53^ The following primers and probes were obtained from Applied Biosystems: IL-1*β*, Mm00434228_m1; GAPDH, Mm99999915_g1.

### Immunocytochemistry

Immunocytochemistry was conducted as previously described. Microglia plated on a glass cover slip were fixed with 4% paraformaldehyde for 10 min. The cells were then permeabilized with 0.05% Triton X-100 for 5 min and blocked with 5% bovine serum albumin for 1 h, followed by incubation with anti-caspase-1 (1 : 500), and anti NLRP3 (1 : 500) antibodies overnight at 4 °C. The cells were then incubated with Alexa 488- or Alexa 568-conjugated secondary antibodies for 1 h. Cells were examined with a deconvolution fluorescence microscope system (Bio Zero, Keyence, Osaka, Japan). Neuronal viability was assessed as previously described.^30, 52^ To determine the viability of neurons in microglia-neuronal cocultures, microglia were labeled with Cy5 conjugated anti-CD11b (1 : 250) for 30 min before permeabilization with 0.05% Triton X-100 for 5 min, and blocked with 5% goat serum for 1 h, followed by incubation with anti-4 G8 antibodies (Chemicon, Temecula, CA, USA, 1 : 1000), and anti-MAP2 antibodies (Merck Millipore, 1 : 1000) for 2 h at room temperature. Then, the cells were incubated with Alexa 488- or Alexa 568-conjugated secondary antibodies (1 : 1000) for 1 h. Cells were examined with a deconvolution fluorescence microscope system.

### Western blotting

Western blotting was done as previously described.^22^ Cell culture supernatants were precipitated by the addition of an equal volume of methanol and 0.25 volumes of chloroform, followed by vortexing and centrifugation for 10 min at 20 000 × *g*. The upper phase was discarded and 500 *μ*l methanol was added to the interphase. This mixture was centrifuged for 10 min at 20 000 × *g*, and the protein pellet was dried and resuspended in Laemmli buffer. The samples were boiled for 5 min at 99 °C. The samples were then separated by SDS-PAGE and transferred onto nitrocellulose membranes. Blots were incubated with rabbit polyclonal anti-mouse caspase-1 antibodies. To determine the caspase-1 level in the cell lysate, microglia were lysed with TNES buffer (1 M Tris-HCL, 20% SDS, and 2.5% glycerol) containing phosphatase (Sigma-Aldrich) and protease inhibitor (Roche, Mannheim, Germany). Fifty micrograms of protein from the total lysate was assayed for caspase-1 and *β*-actin.

### Flow cytometry

Flow cytometry was conducted as previously described.^53^ Briefly, LPS-primed microglia treated with oA*β* or left untreated were stained with 5-(and-6)-chloromethyl–2′,7′-dichlorodihydrofluorescein diacetate, acetyl ester (CM-H_2_DCFDA) or MitoSOX red superoxide indicator (both from Invitrogen, Carlsbad, CA, USA) for 15 min according to the manufacturer's instruction. After washing twice, cells were analyzed using a Cytomics FC500 (Beckman Coulter, Brea, CA, USA).

### Statistical analysis

Statistically significant differences between experimental groups were determined by a one-way ANOVA followed by the Tukey's test for multiple comparisons. Statistical analysis was performed using the software program Prism 4.0 (GraphPad Software, San Diego, CA, USA). *P*-values <0.05 were considered to be statistically significant.

## Abbreviations

AD: Alzheimer's disease
oA*β*: oligomeric amyloid *β*
CNS: central nervous system
IL-1*β*: interleukin-1*β*
NLRP3: NOD-like receptor family, pyrin domain containing 3
ROS: reactive oxygen species
LPS: lipopolysaccharides
APP: amyloid precursor protein
ASC: apoptosis-associated-speck-like protein
NAC: N-acetylcysteine
NOX: NADPH oxidase
