# Supplementary material for: Oligomeric amyloid β induces IL-1β processing via production of ROS: implication in Alzheimer's disease
Source: Cell Death Dis. 2013 Dec 19;4(12):e975–. doi: 10.1038/cddis.2013.503 (PMC3877570; doi:10.1038/cddis.2013.503)
Supplement: Supplementary Figure [file cddis2013503x1.pdf]

Supplementary figure

Fig. 1

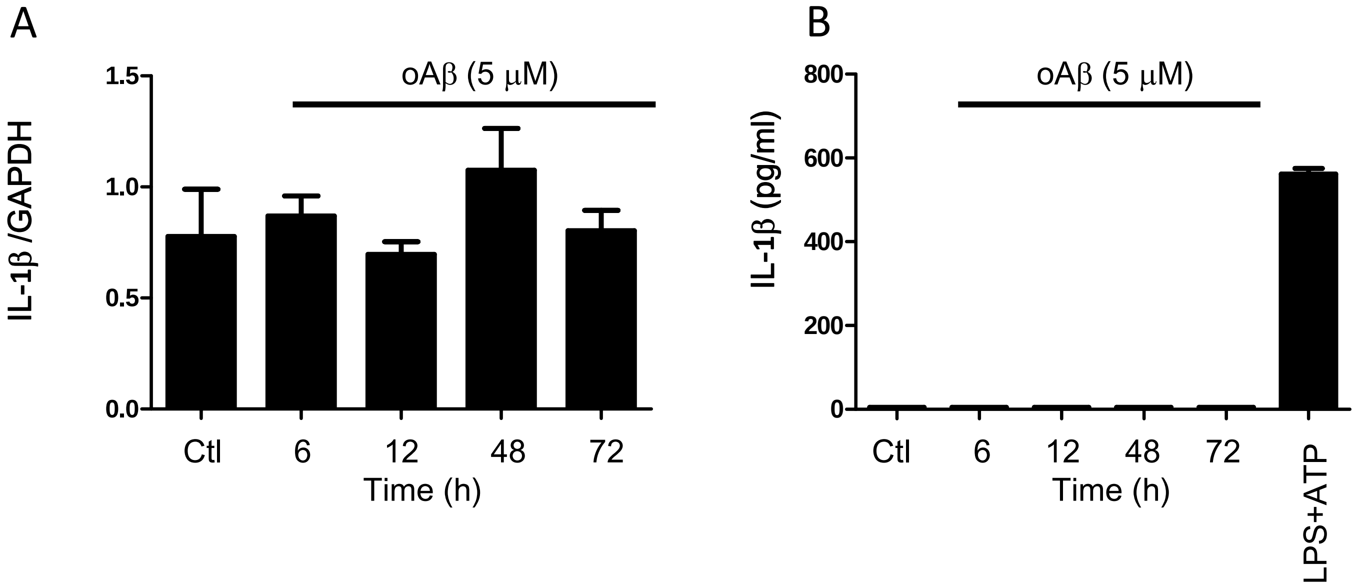

oAβ alone does not induce IL-1β mRNA or protein expression. Microglia treated with oAβ for varying time and the level of IL-1β mRNA as well as concentration of IL-1β in the culture supernatant was measured by qPCR and ELISA(A ,B). Data indicate mean ± SD for five independent experiments.

Fig. 2

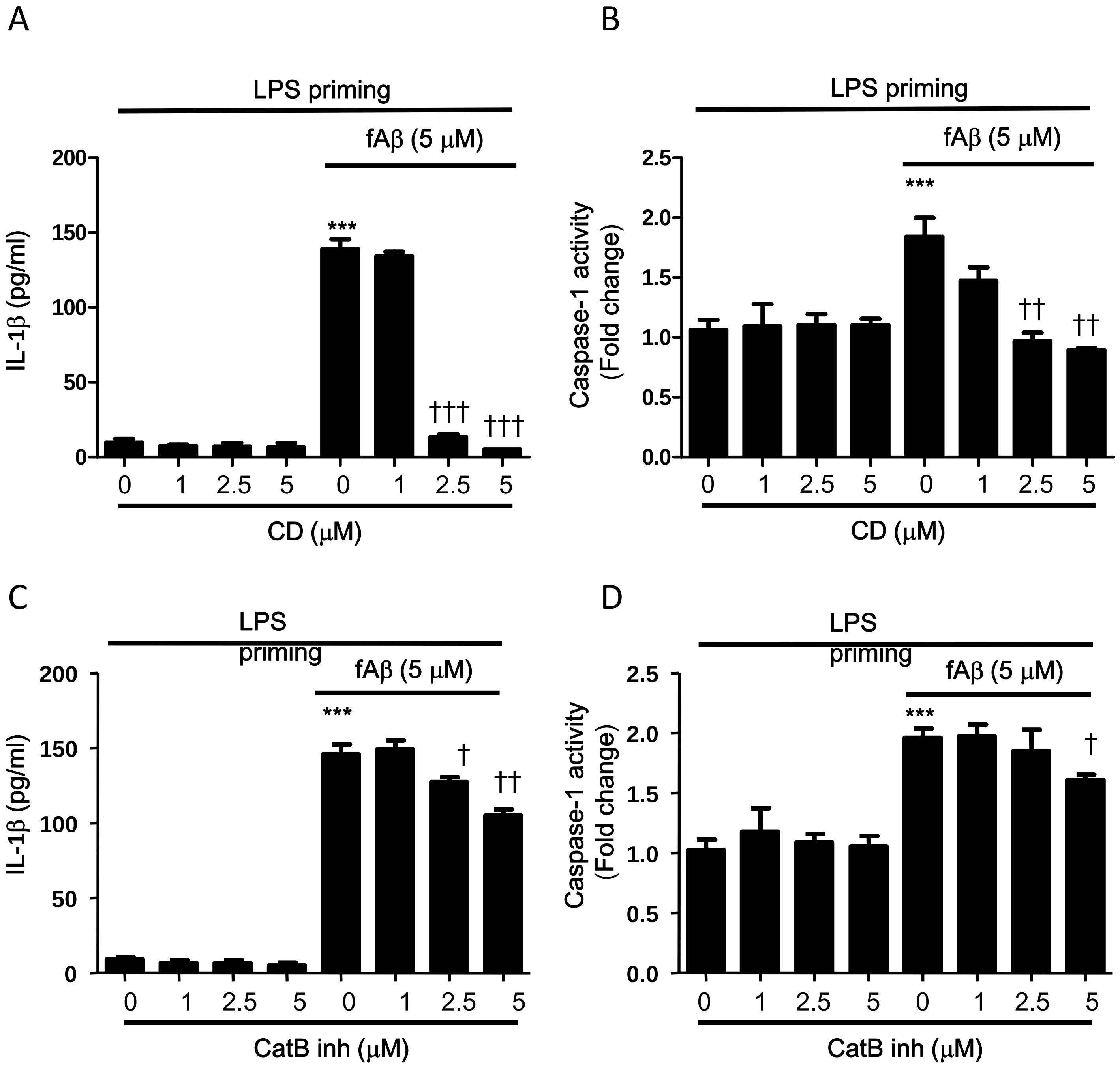

fAβ induced IL-1β release is dependent on phagocytosis and cathepsin B. LPS-primed microglia were treated cytochalasin D (CD) before fAβ stimulation and IL-1β in the culture supernatant (A) as well as caspase-1 activity (B) were measured at 48 h. Data indicate mean ±SD for three independent experiment. ***p < 0.001, versus LPS-primed microglia (ctl). ***p < 0.001, versus LPS-primed microglia (ctl). †, †† p < 0.05, or versus LPS priming + oAβ + CD.

LPS-primed microglia were treated cathepsin B inhibitor (CatB inh) before fAβ stimulation and IL-1β in the culture supernatant (C) as well as caspase-1 activity (D) was measured at 48 h (B). Data indicate mean ±SD for three independent experiment. ***p < 0.001, versus LPS-primed microglia. †, †† p < 0.05, or versus LPS priming + oAβ + Cat B inh. CD indicate cytochalasin D and Cat B inh indicate Cathepsin B inhibitor.

Fig. 3

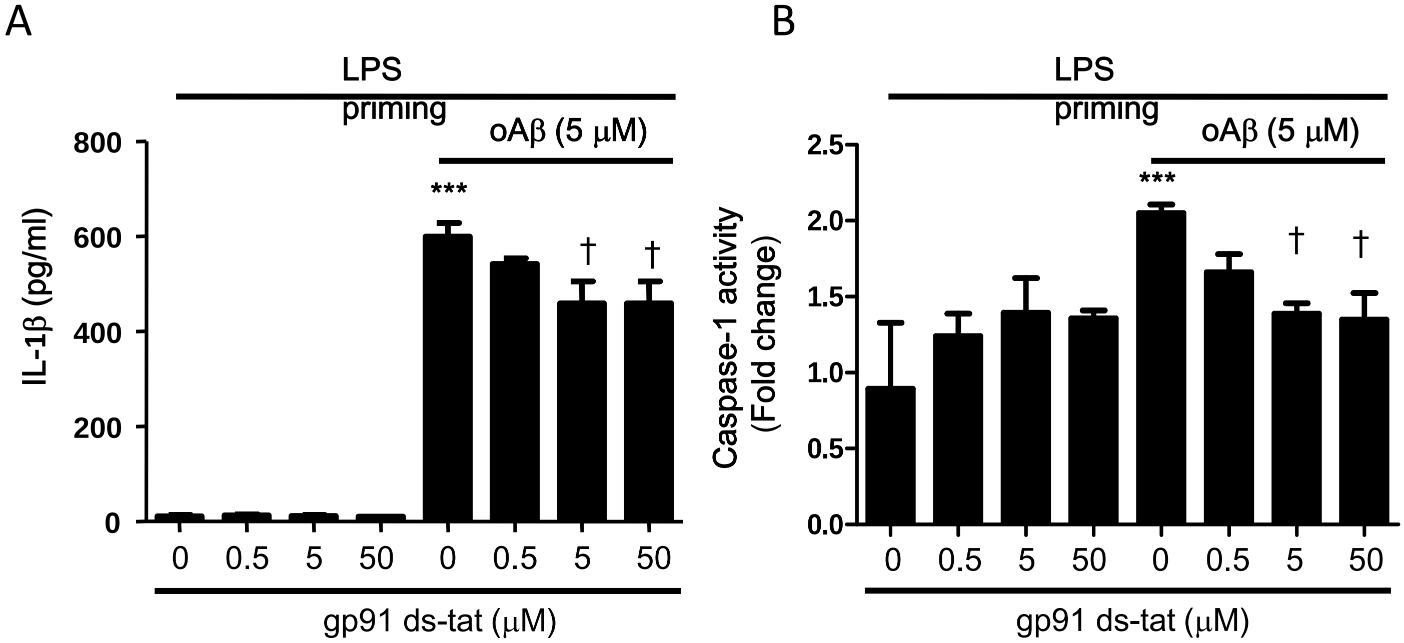

oAβ-induced IL-1β release is partially dependent on NOX2. LPS-primed microglia were treated with gp91 ds-tat for 30 min before oAβ stimulation and IL-1β in the culture supernatant as well as caspase-1 activity was measured at 48 h (A and B). Data indicate mean ±SD for three independent experiment. ***p < 0.001, versus LPS-primed microglia (ctl). ***p < 0.001, versus LPS-primed microglia (ctl). † p < 0.05 versus LPS priming + oAβ + gp91 ds-tat.
